# Projected number of osteoarthritis patients in Austria for the next decades – quantifying the necessity of treatment and prevention strategies in Europe

**DOI:** 10.1186/s12891-022-05091-5

**Published:** 2022-02-09

**Authors:** Wolfgang Hitzl, Tanja Stamm, Margreet Kloppenburg, Markus Ritter, Martin Gaisberger, Antje van der Zee-Neuen

**Affiliations:** 1 Research and Innovation Management (RIM), Biostatistics, Paracelsus Medical University, Salzburg, Austria; 2 Department of Ophthalmology and Optometry, Paracelsus Medical University, Salzburg, Austria; 3 Research Program Experimental Ophthalmology and Glaucoma Research, Paracelsus Medical University, Salzburg, Austria; 4 Section for Outcomes Research, Center for Medical Statistics, Informatics and Intelligent Systems, Medical University of Vienna, Vienna, Austria; 5 Ludwig Boltzmann Institute for Arthritis and Rehabilitation, Medical University of Vienna, Vienna, Austria; 6 Department of Rheumatology, Leiden University Medical Center, Leiden, Netherlands; 7 Center for Physiology, Pathophysiology and Biophysics, Institute for Physiology and Pathophysiology, Salzburg, Austria; 8 Center for Physiology, Pathophysiology and Biophysics, Institute for Physiology, Pathophysiology and Biophysics, Nuremberg, Germany; 9 Ludwig Boltzmann Institute for Arthritis and Rehabilitation, Paracelsus Medical University, Salzburg, Austria; 10 Gastein Research Institute, Paracelsus Medical University, Strubergasse 22, A-5020 Salzburg, Austria; 11 Kathmandu University School of Medical Sciences, Dhulikhel, Nepal; 12 Institute of Nursing Science and Practice, Paracelsus Medical University, Salzburg, Austria; 13 Centre for Public Health and Health Services Research, Paracelsus Medical University, Salzburg, Austria

**Keywords:** Osteoarthritis, Epidemiology, Health economics, Pain assessment and management, Information science, Aging, Greying

## Abstract

**Background:**

The present study aimed to predict the expected number of patients with osteoarthritis (OA) in Austria up to the year 2080.

**Methods:**

Demographic data and population projections between 2019 and 2080 were obtained from European authorities. Information about recent age- and sex-stratified prevalence of patients with self-reported physician-diagnosed OA was obtained from the Austrian Health Interview Survey (*n* = 15,771). Projections were stratified by age and sex; sensitivity analyses were performed based on aging, main (most likely), and growth scenarios of the population.

**Results:**

Based on the projection, the overall increase in the total number of patients with OA from 2019 to 2080 will be 38% for men and women. In 2019, the highest number of OA-patients nested in the groups of persons aged 70-79 (*n* = 238,749) and 60-69 (*n* = 237,729) years. In 2080, the 80+ age group is predicted to have the highest number of OA with 421,548 individuals (i.e. factor 3.45 and factor 2.48 increase in the male and female group, respectively, compared to 2019), followed by the group aged 70-79 with 314,617 individuals (factor 1.45 and factor 1.28 increase in the male and female group, respectively, compared to 2019). Similar trends were found in the ageing and growing scenarios.

**Conclusions:**

The projected increase in the occurrence of OA will likely lead to a substantial socioeconomic burden for the Austrian healthcare system in the near and far future. The current findings plead for the development of sustainable concepts for the treatment and prevention of OA by European authorities.

## Background

Osteoarthritis (OA) is among the most common musculoskeletal diseases in the world, causing pain, loss of function, disability and excess mortality [1, 2]. It can affect all joints, with the highest incidence in weight-bearing joints (i.e. knees and hips) [3]. It is a heterogeneous disorder including several phenotypes and research to establish a unified definition of OA is still ongoing [4]. OA is known to adversely affect (work-)participation resulting in sick leave and work disability, impaired physical functioning in daily life, restrictions in caring for children and relatives, self-care activities, household chores and leisure activities [5, 6]. Hospitalizations due to OA in people aged 50+ already lead to a great impact on public health systems [7–11]. The costs for musculoskeletal disorders, with OA as the most common form, in countries like Australia, Canada, France, United Kingdom and the United States account for 1.0 to 2.5% of the gross domestic product [12, 13]. A Canadian study calculated the direct costs per person and year affected by OA to $3,952 and the indirect costs to $1,760 [14]. A study based on Dutch national data showed that 3-months healthcare costs were 2.3 times higher in persons with musculoskeletal disorders compared to those without. A larger increase in costs was only seen in persons reporting cancer [15].

Up to date, there is no cure for OA. The therapeutic options for OA encompass behavioural, non-pharmacological and/or pharmacological pain-alleviating and functionality improving or stabilizing symptomatic measures as well as surgical measures [2, 16, 17].

Among medications, non-steroidal anti-inflammatory drugs (NSAIDs) are the most frequently used ones, many of them easily available over-the-counter. However, besides their superb effectiveness, NSAIDs have also a broad bandwidth of side effects, especially in patients with comorbidities. They cause gastrointestinal, renal, hepatic, cardiovascular, cerebral and pulmonary complications which not only pose additional harm to the patients but too frequently end up lethally. Around 30% of hospital admissions for cause adverse drug reactions (ADRs) are due to NSAIDs. Likewise, the use of opioid analgesics frequently causes ADRs. This also brings along high bed occupancy and huge costs that add on the socioeconomic impact of OA perse [18–21]. These considerations have also to account for co-medications prescribed to prevent such ADRs, like e.g. anti-secretory drugs (proton pump inhibitors, H2-blockers) to avoid gastrointestinal bleedings [22, 23]. These drugs, though eventually cost effective in a more holistic calculation, may cause ADRs themselves [24].

**Fig. 1:**
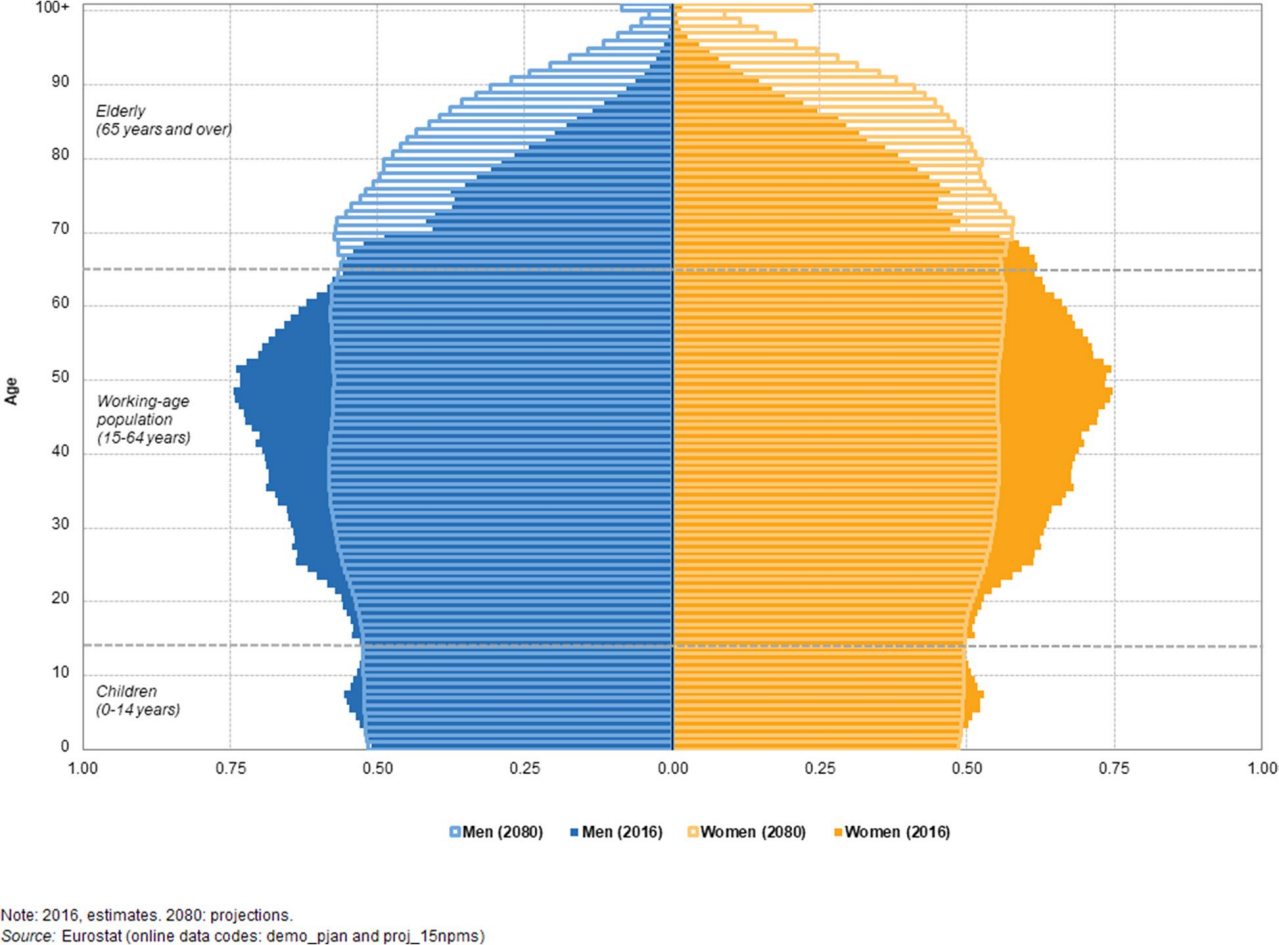
Population pyramids, European Union, 2016 (solid bars) and 2080 (open bars), in percent of the total population, colours: blue, men; orange, women. Reprinted from Eurostat, reuse freely available under citation of original source, figure accessible through https://ec.europa.eu/eurostat/statistics-explained/index.php?title=File:Population_pyramids,_EU-28,_2016_and_2080_(%25_of_total_population)_PITEU17.png&oldid=372204

The increase in life expectancy in the populations of European countries, also known as the “population greying in Europe”, is a demographic phenomenon characterized by a decrease in fertility and an increase of life expectancy. It results in a higher proportion of older people in the (working) society [25]. The comparison of age pyramids for 2016 and 2080 (Fig. 1) shows a continuation of the aging of the EU population. In the coming decades, the number of elderly people will increase significantly. By 2080, the pyramid will develop into the shape of a block, narrowing considerably in the middle of the pyramid (around the age 45–54 years).

In view of this population growth, the growing prevalence of OA with increasing age, its adverse association with work participation, a growing number of the elderly as part of the workforce and the costs related to healthcare consumption (associated with OA-specific complaints or ADRs), it seems plausible to assume that OA will have a growing impact on health care and social systems in Austria and in comparable countries.

Projection of the expected number of OA-patients in the future is a crucial step when planning and budgeting efforts for the treatment and prevention of OA. However, to our best knowledge, no reliable data is available for European countries estimating the number of patients with OAs in the near and distant future. Therefore, the aim of this study was to forecast the number of patients with OA in Austria from 2019 to 2080.

## Methods

### Analytic overview

We used publicly available demographic information and data from the Austrian Health Interview Survey (ATHIS) 2014 questionnaire that estimated the number of subjects with self-reported physician-diagnosed OA from the year 2019 [8]. The survey interviewed 15,771 randomly selected Austrian citizens (i.e. 0.22% of the total general population) aged 15 years or older about their health status and anonymized the data without the possibility of un-blinding. OA was counted if a person stated that this disease occurred within the last 12 months and that it was diagnosed by a physician. The survey only asked for the presence of OA, whereas arthritis (systemic inflammatory form) was explicitly not included in the questioning.

### Demographic data sources

Demographic information on the number of Austrian inhabitants and population projections by decade between 2019 and 2080 was provided by Eurostat.

(https://ec.europa.eu/eurostat/web/main/data/database), the statistical office of the European Union. It provides statistics at the European level that enable comparisons between countries and regions. Specifically, it offers information from economic, demographic, social, ecologic and cultural sectors for the federal and regional authorities as well as for research, socio-economic, and public institutions. Datasets containing demographic information are publicly available and were downloaded from Eurostat (see database: “population and social conditions”, subsection: “population projections”). Data on the demographic development were stratified by age and sex and are given in decades.

### Estimates of age and sex stratified prevalence of patients with self-reported physician-diagnosed OA

Information on age and sex-stratified OA prevalence in Austria among subjects aged ≥20 years (i.e. 12,78 per 100,000 )was obtained from ATHIS 2014 [26] and applied to the year 2019.

### Forecasting the expected number of subjects with OA

Lower and upper bounds for demographic development between 2019 and 2080 were used for three different population scenarios.

- **Aging scenario**: lower fertility, shorter life expectancy, lower rate of immigration.
- **Main scenario**: most likely scenario; mean fertility, mean life expectancy, mean rate of immigration.
- **Growth scenario**: higher fertility, longer life expectancy, higher rate of immigration.

These scenarios are reflecting different assumptions on population growth and aging in Austria deduced from fertility, life expectancy, and immigration calculations which were derived from EUROSTAT scenarios (http://ec.europa.eu/eurostat/data/database, see database: “population and social conditions”, subsection: “population projections”).

“Lower fertility” was defined as 20% lower fertility rates than in the baseline assumptions, in each year of the entire horizon of projections. “Mean fertility” reflected that fertility remained constant over time. “Higher fertility” was defined as 20% higher fertility rates than in the baseline assumptions, in each year of the entire horizon of projections.

“Shorter life expectancy” assumed that the mortality rates are increased such that the life expectancy at birth will decrease by about two years by 2080 when compared with the baseline assumptions. “Longer life expectancy” assumed that the mortality rates are decreased such that the life expectancy at birth will increase by about two years by 2080 when compared with the baseline assumptions.

“Lower rate of immigration” was defined as 33% lower net migration than in the baseline assumptions, in each year of the entire horizon of projections. A “higher rate of immigration” reflected that the net migration was 33% higher than in the baseline assumptions, in each year of the entire horizon of projections.

The forecasts for OA total numbers were created under the assumption that the prevalence of OA would stay stable within each age and sex group during the investigated period. Based on the changing absolute number of people within each stratum, the number of patients with OA either increases or decreases. Computation was done by combining population projections provided by Eurostat with the assumption of stable prevalence over time. For example, in 2019, the prevalence of OA among women aged 70-75 was 41.025% (86,520/210,892). The absolute number of women in this age category is expected to increase to 278,189 by the year 2080, and thus we estimated the number of women with OA aged 70-75 to be 114,129 in 2080 (i.e. 278,189*0.41025). Predictions were carried out for the three different population scenarios and were stratified by age and sex. The age groups studied were: 20-29, 30-39, 40-49,50-59, 60-69, 70-79 and 80+ years, respectively.

For the main-scenario, the sex-specific prevalence of OA was projected per 5-year increments (i.e. 2019-2080).

## Results

In 2019, the total Austrian population aged 20+ years consisted of 7.16 million people (3.48 million men and 3.68 million women, respectively). Using the ageing scenario resulted in an estimated population of 8.1 million people in 2080, while using the growing scenario predicted a population of 9.97 million people.

**Fig. 2:**
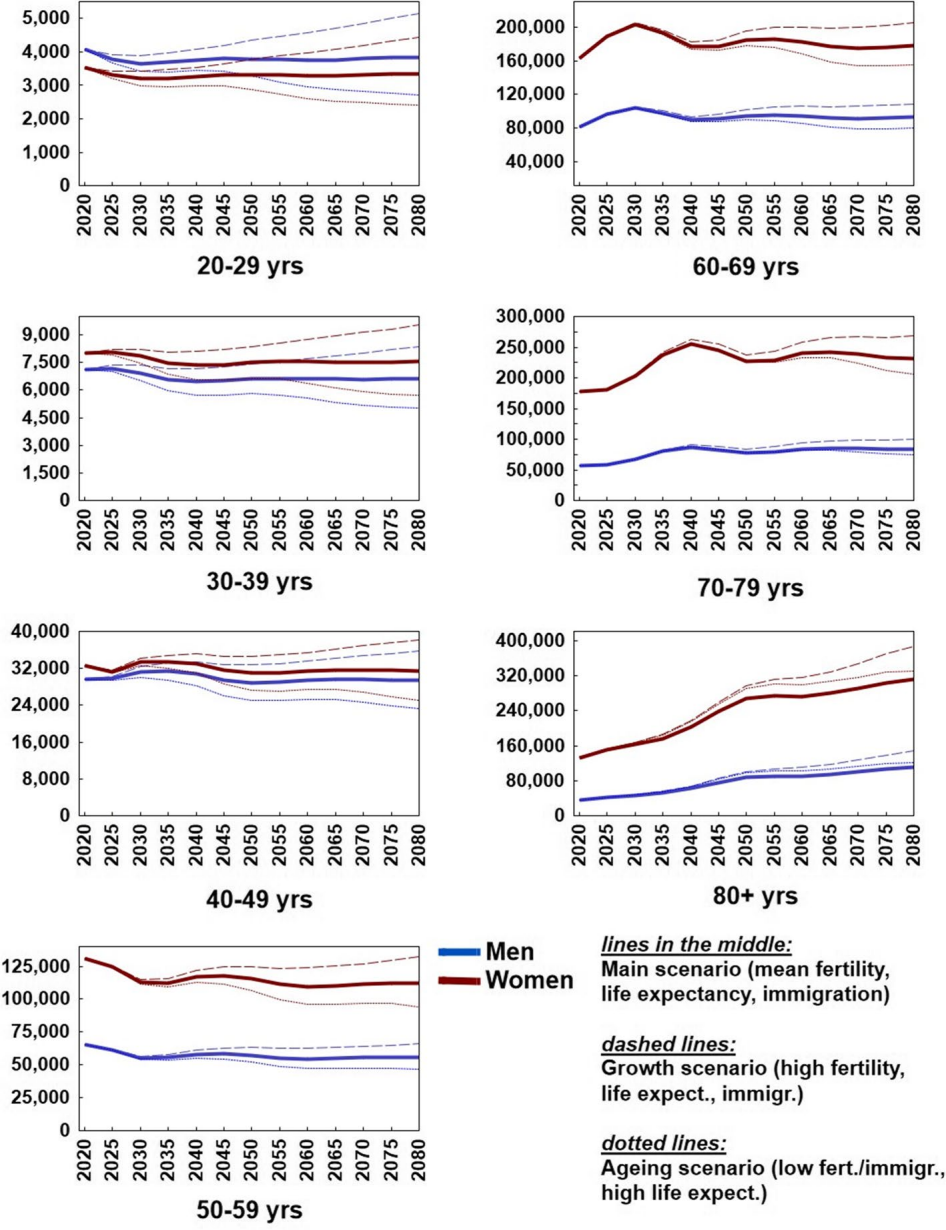
Age-dependent prevalence of self-reported physician-diagnosed osteoarthritis in Austria in 2019 stratified by age and sex. Source-data derived from the Austrian Health Interview Survey

**Fig. 3:**
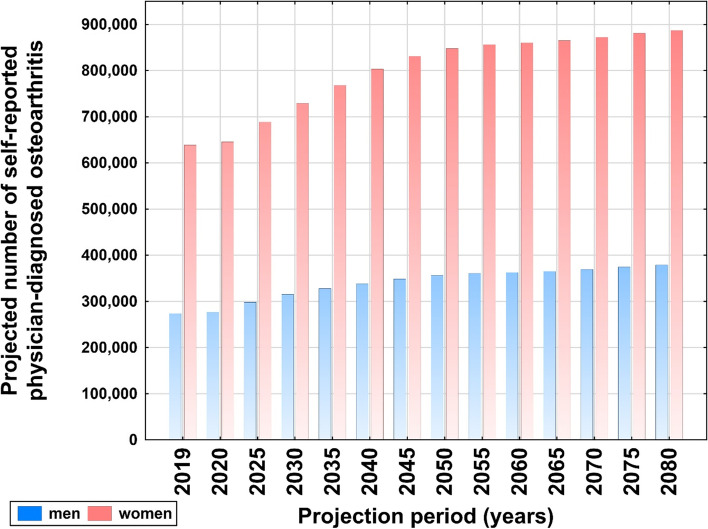
Sex-stratified projected number of self-reported physician-diagnosed osteoarthritis in Austria from 2019-2080 per 5 year increment sex (main scenario). Source-data derived from the Austrian Health Interview Survey

Approximately 13% (12.78%, n = 914,652) of the Austrian population reported physician-diagnosed OA in 2019 [8% (7.88%, n = 247,599) of all men ≥20 years and 17% (17.41%, n = 640,054) of all women]. A detailed overview of the age- and sex-stratified prevalence is provided in Fig. 2. The sex-specific prevalence of OA per 5-year increment for the main scenario is provided in Fig. 3.

All scenarios predicted considerably higher numbers of OA-patients in 2080. Table 1 shows the estimated percent change in the total number of subjects with self-reported physician-diagnosed OA from 2019 to 2080 for each of the population growth scenarios (i.e. main scenario of population, growing scenario and ageing scenario).

**Table 1:**
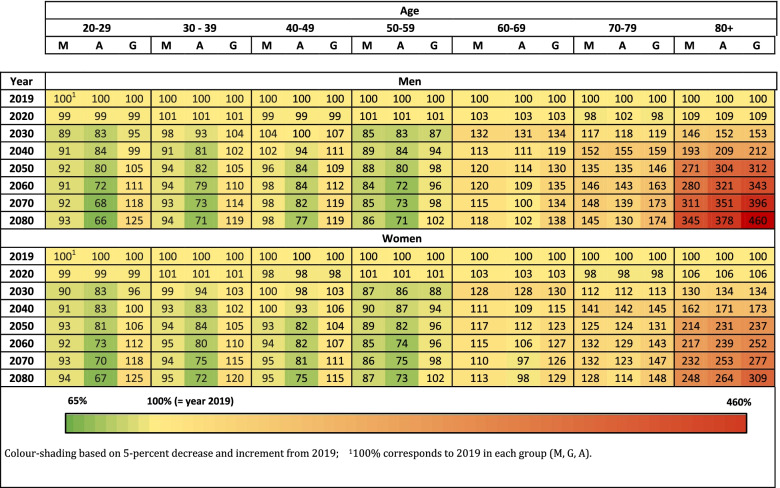
Estimated change (%) of subjects with self-reported physician-diagnosed osteoarthritis from 2019 to 2080 in Austria. Estimates are based on the main scenario of population growth (M), growing scenario (G) and ageing scenario (A)

Using the main scenario, the population of all subjects aged 20 years and older was estimated to increase to 8.08 million in the year 2080 (3.94 million men and 4.13 million women; i.e. +13% for both sexes). From 2019 to 2080 the corresponding number of subjects reporting physician-diagnosed osteoarthritis was estimated to increase by 38%. The total number of Austrian subjects reporting physician-diagnosed OA in the year 2019 was 12,775 per 100,000 individuals with more women than men affected (i.e. 640,054 women compared to 274,599 men in absolute numbers). The forecast predicted 15,570 per 100,000 individuals aged 20+ for the year 2080 (i.e. 876,426 women and 382,437 men).Of all subjects younger than 20 years of age 0.36% of had self-reported physician-diagnosed osteoarthritis (data not shown).

Aged-stratified estimations showed alterations in the age group most affected by OA: In 2019, the group with the highest numbers of OA patients was 70-79 years of age (238,749 individuals), followed by the 60-69 years group (237,729 individuals). For 2080, the forecast predicted that the age group 80+ will comprise the highest number of OA patients (421,548 individuals), followed by the 70-79 years group (314,617 individuals) (Fig. 2).

From 2019 to 2080, the number of OA patients in the age groups 60-69, 70-79 and 80+ were estimated to increase by factors 1.18, 1.45 and 3.45 in men and 1.13, 1.28 and 2.48 in women, respectively.

**Fig. 4:**
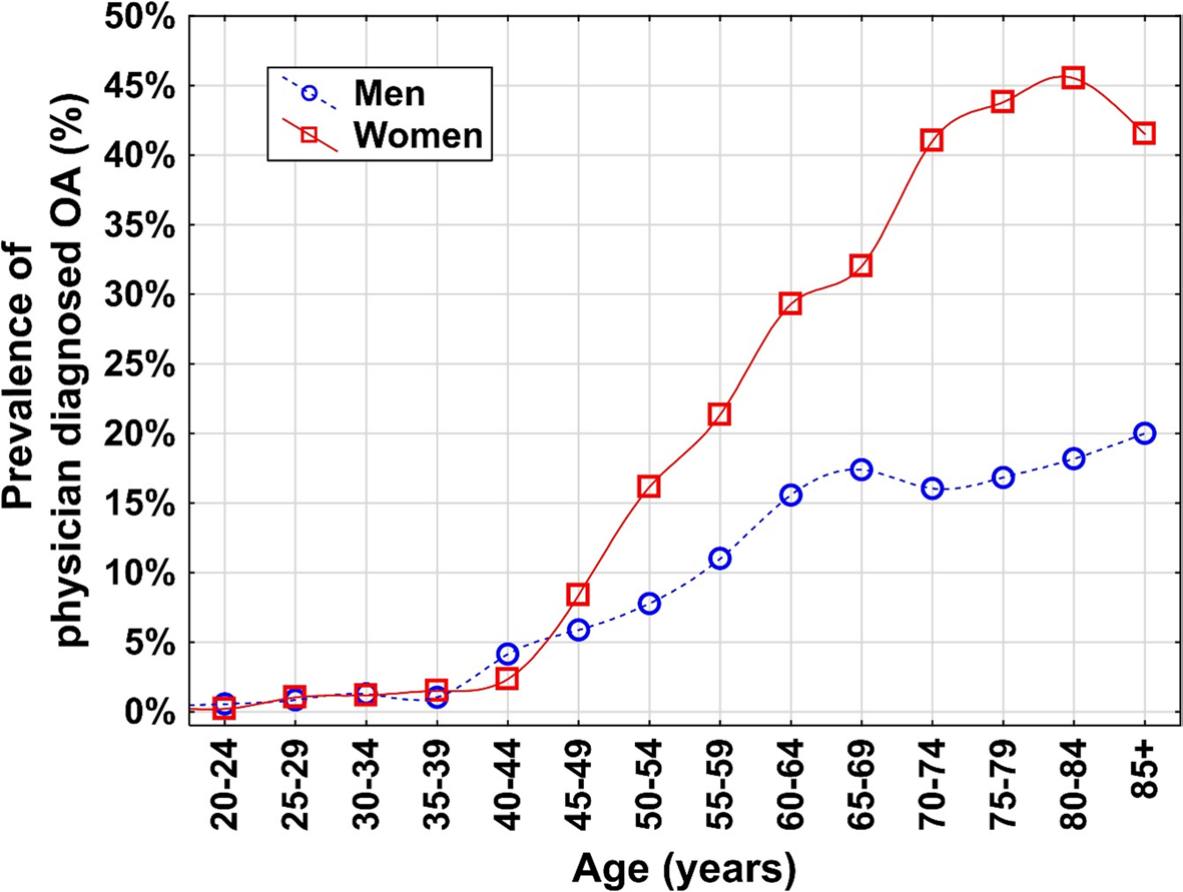
Estimated prevalence of self-reported physician-diagnosed osteoarthritis in Austria for men in women in all age-groups based on 2019 prevalence estimates from the Austrian Health Interview Survey (ATHIS)

The projected number of subjects with self-reported physician-diagnosed OA in Austria from 2019 to 2080 for all age and sex strata is illustrated in Fig. 4.

## Discussion

The results of the current study reveal that the total number of persons with OA in Austria will increase substantially until the year 2080. The overall predicted increase is 38% when comparing results for 2019 and 2080 in men and women. The largest increase is seen in the male groups aged 70-79 and 80+, which showed an increase of 45% and 245%, respectively. In comparison, women in these two age groups are estimated to contribute to the increase with 28% and 148%. This forecast is based on the estimated main scenario. A calculation of a growth scenario (i.e. high fertility, long life expectancy, and an additional high rate of immigration), results in an expected increase of 360% for men and 209% for women aged 80+ from 2019 to 2080. Considering the current immigration situation in Europe, the latter scenario seems most plausible. During the last decades life expectation in western countries rose constantly and the gap in average mortality age between men and women is getting smaller [27]. Considering enhanced medical treatment and stable political systems, it is plausible to assume that by the year 2080 far more men will reach 80+ years. According to Eurostat, the European population aged 75+ years will increase between 160 and 476% (numbers from Italy and Luxemburg, respectively) depending on which European country. An age and sex stratified constant OA prevalence will therefore increase the total number of OA patients severely also due to the fact that the prevalence is highly age-dependent.

In accordance with our own findings, a Swedish population-based study projected an increase in OA prevalence among adults aged 45 and older from 26.6% to 2012 to 29.5% in 2032 [28]. An Australian study predicted a steep increase in OA-prevalence as well showing an increase from 2.2 million OA patients in 2015 to almost 3.1 million in 2030. The study also stated that OA associated healthcare costs would increase from over $2.1 billion in 2015 to more than $2.9 billion in 2030 ($970 for every OA-patient) [29]. This increase in prevalence and costs over a relatively short period of time points towards the relevant impact the increase may have on healthcare systems when considering a longer period of time.

### Limitations and considerations

The present study is based on demographic estimates for the period from 2019 to 2080 in the Austrian population. Naturally, factors such as migration, population growth, and aging deduced from fertility, life expectancy, and immigration calculations may vary considerably until 2080. To account for these factors, we provided three different scenarios, i.e. an aging, main (most likely) and growth scenario, respectively. Socio-economic changes of the population, war, and natural disasters were assumed to remain unaltered during the projection period. Though this approach has restrictions, it nevertheless allows for the application of the forecast model in the calculation of age-adjusted predictions of OA total numbers in other western countries if adjusted to the respective population growth scenarios. The current analyses are exclusively reflections of the demographic changes of the Austrian population. Changes in actual OA prevalence rates were not assumed due to the complex nature of potential factors influencing the actual increase or decrease of the occurrence of OA. Changes in environmental influences on the population like increase in health literacy, increase in mobility/migration, natural disasters or war were not taken into account. On the one hand, changes in environmental influences on the population, smart homes, work behaviour and assistive technologies may increase OA prevalence due to an increase in body mass index (BMI) [30, 31]. On the other hand, improved prevention, increased health literacy, more physical activity and better medical treatment may decrease the prevalence [32–34]. However, research regarding these preventive measures is still extremely limited. Therefore, future effects of these factors on OA prevalence cannot be reliably estimated at the moment. Age and BMI adjusted comparisons between early and post-industrial samples show at least a 2.1 times higher prevalence of knee OA in the United States of America [35]. This indicates that there are more, yet not entirely explored risk factors playing a role in the prevalence of OA. A more precise estimation of joints affected by OA could be useful when designing preventive strategies. Therefore, future health surveys should ideally discriminate OA in subgroups like knee, hip, spine and hand. Epidemiologic studies show that the prevalence of OA in the western world is around 14.8% (n = 4733; age ≥ 18 years) [36]. Estimates of the American Bone and Joint Initiatives show that almost 23% of the US population report a physician diagnosed form of arthritis with OA representing the largest part of this group [37]. Our number of self-reported physician diagnosed OA is in good accordance with these numbers and indicates that the data is reliable. While diagnosis by health professionals is certainly most reliable, previous research showed that asking respondents specifically for physician-confirmed diagnosis is beneficial in increasing the accuracy of self-reported disease [38].

## Conclusions

The increase in osteoarthritis patients predicted by the current study emphasizes the necessity of developing, implementing and financing sustainable interventions for the treatment but - more importantly - the prevention of osteoarthritis. Authority-driven public health campaigns might use these numbers to inform community policy makers and general populations and to initiate lifestyle changes promoting healthy behaviour aiming to reduce the incidence of OA.

## Data Availability

The datasets used and/or analysed during the current study are available from the corresponding author on reasonable request. Projection data were obtained via the European Commission database Eurostat http://ec.europa.eu/eurostat/data/database. ATHIS data was obtained via Statistics Austria: http://www.statistik.at/web_de/services/mikrodaten_fuer_forschung_und_lehre/datenangebot/standardisierte_datensaetze_sds/index.html.

## Abbreviations

ATHIS: Austrian Health Interview Survey
BMI: body mass index
EU: European Union
OA: osteoarthritis
